# 2-(4-Meth­oxy­phen­yl)-1-pentyl-4,5-di­phenyl-1*H*-imidazole

**DOI:** 10.1107/S1600536812049100

**Published:** 2012-12-05

**Authors:** Jim Simpson, Shaaban K. Mohamed, Adel A. Marzouk, Avtandil H. Talybov, Antar A. Abdelhamid

**Affiliations:** a Department of Chemistry, University of Otago, PO Box 56, Dunedin, New Zealand; b Chemistry Department, Faculty of Science, Minia University, El-Minia, Egypt; c Chemistry and Environmental Division, Manchester Metropolitan University, Manchester M1 5GD, England; d Pharmaceutical Chemistry Department, Faculty of Pharmacy, Al Azhar University, Egypt; e Manedaliev Institute of Petrochemical Processes, National Academy of Sciences of Azerbaijan, Baku, Azerbaijan; f Chemistry Department, Faculty of Science, Sohag University, Sohag 82524, Egypt

## Abstract

The title compound, C_27_H_28_N_2_O, is a lophine (2,4,5-triphenyl-1*H*-imidazole) derivative with an *n*-pentyl chain on the amine N atom and a 4-meth­oxy substituent on the benzene ring. The two phenyl and meth­oxy­benzene rings are inclined to the imidazole ring at angles of 25.32 (7), 76.79 (5) and 35.42 (7)°, respectively, while the meth­oxy substituent lies close to the plane of its benzene ring, with a maximum deviation of 0.126 (3) Å for the meth­oxy C atom. In the crystal, inversion dimers linked by pairs of C—H⋯O hydrogen bonds generate *R*_2_^2^(22) loops. These dimers are stacked along the *a-*axis direction.

## Related literature

For the non-linear optical and chemiluminescence properties of lophine and its derivatives, see: Santos *et al.* (2001 ▶); Radziszewski (1877 ▶); Maeda & Hayashi (1969 ▶, 1970 ▶). For the bioactivity of imidazoles, see: Antolini *et al.* (1999 ▶); Eyers *et al.* (1998 ▶); Laszlo *et al.* (1999 ▶); Newman *et al.* (2000 ▶); Veisi *et al.* (2012 ▶); Wang *et al.* (2002 ▶). For related structures, see, for example: Yanover & Kaftory (2009*a* ▶,*b* ▶); Kison & Opatz (2009 ▶); Zhao *et al.* (2012 ▶). For representative bond lengths, see: Allen *et al.* (1987 ▶) and for hydrogen-bond motifs, see: Bernstein *et al.* (1995 ▶).
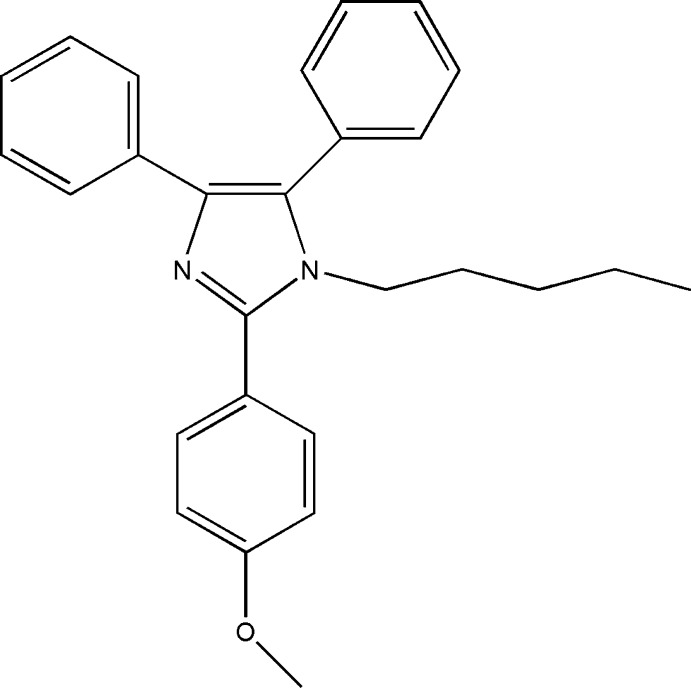


## Experimental

### 

#### Crystal data

C_27_H_28_N_2_O
*M* *_r_* = 396.51
Triclinic, 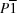

*a* = 9.7214 (19) Å
*b* = 10.739 (1) Å
*c* = 11.7367 (10) Å
α = 114.069 (4)°
β = 99.021 (6)°
γ = 95.425 (6)°
*V* = 1087.7 (3) Å^3^
*Z* = 2
Mo *K*α radiation
μ = 0.07 mm^−1^
*T* = 93 K
0.47 × 0.18 × 0.08 mm

#### Data collection

Bruker APEXII CCD area-detector diffractometer
Absorption correction: multi-scan (*SADABS*; Bruker, 2011 ▶) *T*_min_ = 0.619, *T*_max_ = 0.746
15574 measured reflections
4968 independent reflections
3542 reflections with *I* > 2σ(*I*)
*R*_int_ = 0.043

#### Refinement

*R*[*F* ^2^ > 2σ(*F* ^2^)] = 0.048
*wR*(*F* ^2^) = 0.132
*S* = 1.06
4968 reflections
273 parameters
H-atom parameters constrained
Δρ_max_ = 0.21 e Å^−3^
Δρ_min_ = −0.25 e Å^−3^

### 

Data collection: *APEX2* (Bruker, 2011 ▶); cell refinement: *APEX2* and *SAINT* (Bruker, 2011 ▶); data reduction: *SAINT* (Bruker, 2011 ▶); program(s) used to solve structure: *SHELXS97* (Sheldrick, 2008 ▶) and *TITAN2000* (Hunter & Simpson, 1999 ▶); program(s) used to refine structure: *SHELXL97* (Sheldrick, 2008 ▶) and *TITAN2000*; molecular graphics: *SHELXTL* (Sheldrick, 2008 ▶) and *Mercury* (Macrae *et al.*, 2008 ▶); software used to prepare material for publication: *SHELXL97*, *enCIFer* (Allen *et al.*, 2004 ▶), *PLATON* (Spek, 2009 ▶) and *publCIF* (Westrip, 2010 ▶).

## Supplementary Material

Crystal structure: contains datablock(s) global, I. DOI: 10.1107/S1600536812049100/bt6874sup1.cif

Structure factors: contains datablock(s) I. DOI: 10.1107/S1600536812049100/bt6874Isup2.hkl

Supplementary material file. DOI: 10.1107/S1600536812049100/bt6874Isup3.cml

Additional supplementary materials:  crystallographic information; 3D view; checkCIF report

## Figures and Tables

**Table 1:** Hydrogen-bond geometry (Å, °)

*D*—H⋯*A*	*D*—H	H⋯*A*	*D*⋯*A*	*D*—H⋯*A*
C15—H15⋯O119^i^	0.95	2.61	3.4393 (19)	146

Symmetry code: (i) 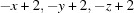.

**Fig. 1.**
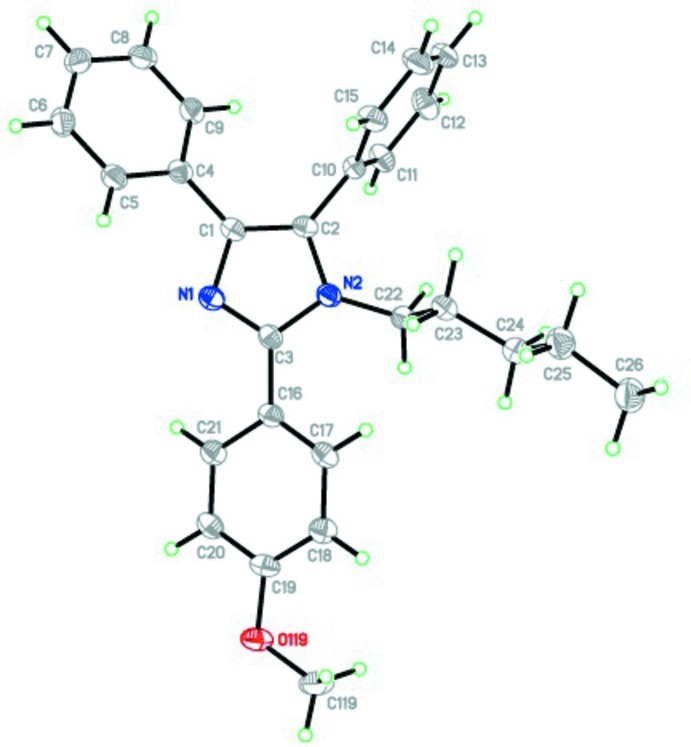
The structure of I with ellipsoids drawn at the 50% probability level.

**Fig. 2.**
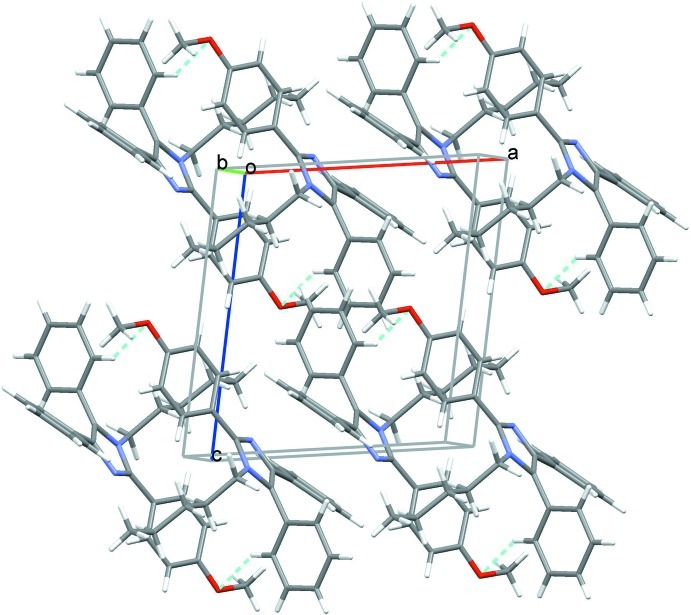
Crystal packing of I viewed along b showing centrosymmetric dimer formation. Hydrogen bonds are drawn as dashed lines.

**Fig. 3.**
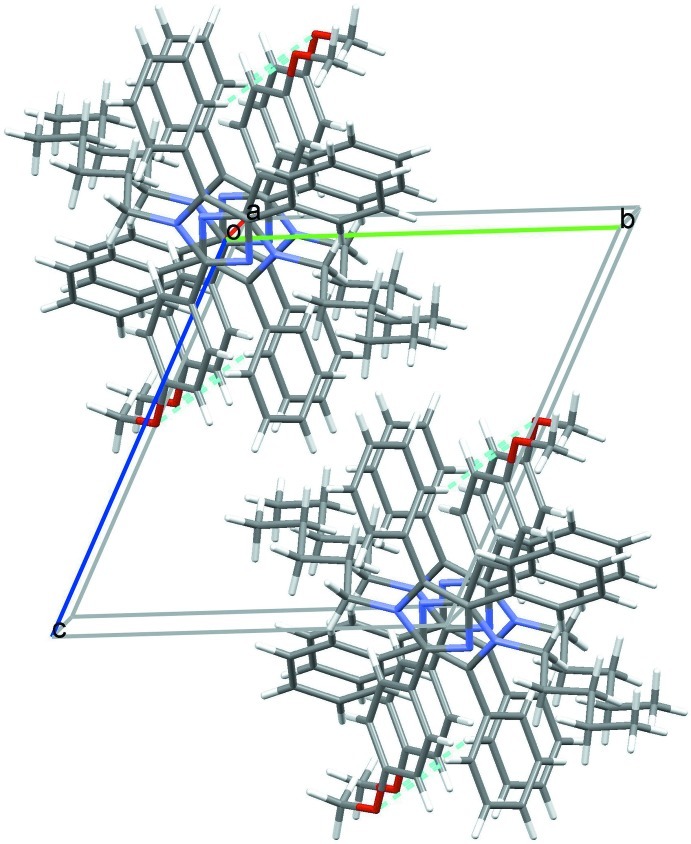
Crystal packing of I showing stacks formed along a. Hydrogen bonds are drawn as dashed lines.

## supplementary crystallographic information

### Comment

The chemilumescence properties of lophine, (2,4,5-triphenyl-1*H*-imidazole), and its derivatives have been known since the late 19t h century (Radziszewski, 1877) and their non-linear optical (Santos *et al.*, 2001) and related optical properties (Maeda & Hayashi, 1969, 1970) have been extensively investigated. In addition, substituted imidazoles exhibit a wide range of biological activities for example as glucagon receptors (Laszlo *et al.*, 1999), CB1 cannabinoid receptor antagonists (Eyers *et al.*, 1998) and modulators of P-glycoprotein (P-gp)-mediated multi drug resistance (MDR) (Newman *et al.*, 2000). They can also act as both antibacterial (Antolini *et al.*, 1999) and antitumor agents (Wang *et al.*, 2002) or as pesticides (Veisi *et al.*, 2012). As part of our work on the synthesis of imidazole derivatives, we have prepared 2-(4-methoxyphenyl)-1-pentyl-4,5-diphenyl-1*H*-imidazole and report its preparation and structure here.

In the title compound, the lophine (2,4,5-triphenyl-1*H*-imidazole) skeleton (Yanover & Kaftory, 2009*a*) is embellished with a nicely ordered C22—C26 *n*-pentyl substituent on the amine N atom of the imidazole ring and a *p*-methoxy substituent on the C17—C21 benzene ring. The *n*-pentyl chain is almost orthogonal to the imidazole with a meanplane through C22···C26 (r.m.s. deviation = 0.047 /%A) that subtends a dihedral angle of 78.91 (7) ° to the plane of the imidazole ring. The two C4···C9 and C10···C15 phenyl rings are inclined to the imidazole ring at angles of 25.32 (7)°, 76.79 (5)° respectively while the methoxy substituted C17···C21 ring makes and angle of 35.42 (7)°. The methoxy substituent lies close to the plane of the C17···C21 benzene ring with a maximum deviation of only 0.126 (3) Å for the C119 atom. Bond distances in the structure are normal (Allen *et al.*, 1987) and are comparable to those reported for related structures (Yanover & Kaftory, 2009*a*,*b*; Kison & Opatz, 2009; Zhao *et al.*, 2012). In the crystal structure the only significant intermolecular contacts are C15—H15···O119 hydrogen bonds which form inversion dimers with *R*_2_^2^(22) ring motifs (Bernstein *et al.*, 1995). These dimers are further stacked along the *a* axis (Fig. 2), with alternating molecules arranged in a head to tail fashion (Fig. 3).

### Experimental

A 50-ml. volumetric flask equipped with a magnetic stirring bar was charged with 25 ml. of dimethyl sulfoxide and 2.4 g (40 mmol) potassium hydroxide. The mixture was stirred at room temperature for 5 minutes, then 3.26 g (10 mmol) 2-(4-methoxyphenyl)-4,5-diphenyl-1*H*-imidazole was added with stirring for a further 45 minutes. To this reaction mixture, 3.02 g. (20 mmol) pentyl bromide was added. After stirring for an additional 45 minutes the mixture was diluted with 20 ml water then extracted with diethyl ether (3x 20 ml). The combined ether layers were dried over calcium chloride and evaporated under slightly reduced pressure. The excess pentyl bromide was removed by distillation at approximately 15 mm, and the residue was crystallized from ethanol yielding 3.35 g (84%) of 2-(4-methoxyphenyl)-1-pentyl-4,5-diphenyl-1*H*-imidazole, m.p. 382–384 K.

### Refinement

All H-atoms bound were refined using a riding model with d(C—H) = 0.95 Å, *U*_iso_=1.2*U*_eq_ (C) for aromatic, 0.99 Å *U*_iso_=1.2*U*_eq_ (C) for methylene and 0.98 Å, *U*_iso_ = 1.5*U*_eq_ (C) for CH_3_ H atoms.

### Crystal data

**Table:**

C_27_H_28_N_2_O	*Z* = 2
*M**_r_* = 396.51	*F*(000) = 424
Triclinic, *P*1	*D*_x_ = 1.211 Mg m^−^^3^
Hall symbol: -P 1	Mo *K*α radiation, λ = 0.71073 Å
*a* = 9.7214 (19) Å	Cell parameters from 3238 reflections
*b* = 10.739 (1) Å	θ = 2.6–26.1°
*c* = 11.7367 (10) Å	µ = 0.07 mm^−^^1^
α = 114.069 (4)°	*T* = 93 K
β = 99.021 (6)°	Rectangular plate, colourless
γ = 95.425 (6)°	0.47 × 0.18 × 0.08 mm
*V* = 1087.7 (3) Å^3^	

### Data collection

**Table:**

Bruker APEXII CCD area-detector diffractometer	4968 independent reflections
Radiation source: fine-focus sealed tube	3542 reflections with *I* > 2σ(*I*)
Graphite monochromator	*R*_int_ = 0.043
ω scans	θ_max_ = 27.6°, θ_min_ = 3.1°
Absorption correction: multi-scan (*SADABS*; Bruker, 2011)	*h* = −12→12
*T*_min_ = 0.619, *T*_max_ = 0.746	*k* = −13→14
15574 measured reflections	*l* = −15→15

### Refinement

**Table:**

Refinement on *F*^2^	Primary atom site location: structure-invariant direct methods
Least-squares matrix: full	Secondary atom site location: difference Fourier map
*R*[*F*^2^ > 2σ(*F*^2^)] = 0.048	Hydrogen site location: inferred from neighbouring sites
*wR*(*F*^2^) = 0.132	H-atom parameters constrained
*S* = 1.06	*w* = 1/[σ^2^(*F*_o_^2^) + (0.0663*P*)^2^ + 0.0245*P*] where *P* = (*F*_o_^2^ + 2*F*_c_^2^)/3
4968 reflections	(Δ/σ)_max_ < 0.001
273 parameters	Δρ_max_ = 0.21 e Å^−^^3^
0 restraints	Δρ_min_ = −0.25 e Å^−^^3^

### Special details

**Table:**

Geometry. All e.s.d.'s (except the e.s.d. in the dihedral angle between two l.s. planes) are estimated using the full covariance matrix. The cell e.s.d.'s are taken into account individually in the estimation of e.s.d.'s in distances, angles and torsion angles; correlations between e.s.d.'s in cell parameters are only used when they are defined by crystal symmetry. An approximate (isotropic) treatment of cell e.s.d.'s is used for estimating e.s.d.'s involving l.s. planes.
Refinement. Refinement of *F*^2^ against ALL reflections. The weighted *R*-factor *wR* and goodness of fit *S* are based on *F*^2^, conventional *R*-factors *R* are based on *F*, with *F* set to zero for negative *F*^2^. The threshold expression of *F*^2^ > σ(*F*^2^) is used only for calculating *R*-factors(gt) *etc*. and is not relevant to the choice of reflections for refinement. *R*-factors based on *F*^2^ are statistically about twice as large as those based on *F*, and *R*- factors based on ALL data will be even larger.

### Fractional atomic coordinates and isotropic or equivalent isotropic displacement parameters (Å^2^)

**Table:**

	*x*	*y*	*z*	*U*_iso_*/*U*_eq_	
C1	0.65447 (14)	1.04609 (14)	0.93573 (12)	0.0209 (3)	
C2	0.63626 (14)	0.90460 (14)	0.87092 (12)	0.0208 (3)	
N2	0.73101 (12)	0.86376 (11)	0.94408 (10)	0.0214 (3)	
C3	0.80178 (14)	0.98139 (14)	1.04912 (13)	0.0206 (3)	
N1	0.75725 (12)	1.09265 (12)	1.04654 (10)	0.0218 (3)	
C4	0.58701 (15)	1.14575 (14)	0.89992 (12)	0.0213 (3)	
C5	0.65877 (16)	1.28159 (15)	0.94687 (13)	0.0252 (3)	
H5	0.7490	1.3086	1.0031	0.030*	
C6	0.60042 (16)	1.37763 (15)	0.91278 (14)	0.0276 (3)	
H6	0.6507	1.4696	0.9454	0.033*	
C7	0.46848 (16)	1.33933 (16)	0.83088 (14)	0.0290 (4)	
H7	0.4288	1.4045	0.8064	0.035*	
C8	0.39535 (17)	1.20596 (16)	0.78529 (14)	0.0283 (4)	
H8	0.3045	1.1798	0.7302	0.034*	
C9	0.45371 (15)	1.11015 (15)	0.81940 (13)	0.0244 (3)	
H9	0.4022	1.0188	0.7875	0.029*	
C10	0.54830 (15)	0.80462 (14)	0.74497 (13)	0.0210 (3)	
C11	0.42624 (16)	0.71869 (15)	0.73438 (14)	0.0279 (4)	
H11	0.3970	0.7226	0.8091	0.033*	
C12	0.34667 (17)	0.62676 (16)	0.61431 (15)	0.0334 (4)	
H12	0.2637	0.5677	0.6077	0.040*	
C13	0.38700 (17)	0.62053 (16)	0.50497 (14)	0.0310 (4)	
H13	0.3321	0.5577	0.4233	0.037*	
C14	0.50733 (17)	0.70598 (16)	0.51489 (14)	0.0329 (4)	
H14	0.5353	0.7026	0.4398	0.040*	
C15	0.58783 (16)	0.79690 (15)	0.63386 (13)	0.0280 (4)	
H15	0.6713	0.8548	0.6396	0.034*	
C16	0.90797 (15)	0.98543 (14)	1.15551 (13)	0.0216 (3)	
C17	1.00822 (15)	0.89913 (15)	1.14112 (13)	0.0240 (3)	
H17	1.0102	0.8329	1.0579	0.029*	
C18	1.10545 (15)	0.90705 (15)	1.24484 (13)	0.0249 (3)	
H18	1.1723	0.8462	1.2326	0.030*	
C19	1.10445 (15)	1.00433 (15)	1.36650 (13)	0.0243 (3)	
O119	1.19783 (10)	1.02238 (11)	1.47489 (9)	0.0288 (3)	
C119	1.28969 (17)	0.92355 (17)	1.46185 (15)	0.0351 (4)	
H11A	1.2332	0.8303	1.4256	0.053*	
H11B	1.3471	0.9442	1.5459	0.053*	
H11C	1.3518	0.9283	1.4050	0.053*	
C20	1.00777 (15)	1.09470 (15)	1.38280 (13)	0.0255 (3)	
H20	1.0087	1.1633	1.4658	0.031*	
C21	0.91080 (15)	1.08526 (15)	1.27931 (13)	0.0242 (3)	
H21	0.8450	1.1471	1.2918	0.029*	
C22	0.75400 (15)	0.72148 (14)	0.90983 (13)	0.0228 (3)	
H22A	0.6625	0.6574	0.8669	0.027*	
H22B	0.7908	0.7114	0.9886	0.027*	
C23	0.85838 (15)	0.68150 (14)	0.82145 (13)	0.0241 (3)	
H23A	0.8103	0.6648	0.7344	0.029*	
H23B	0.9379	0.7599	0.8518	0.029*	
C24	0.91712 (16)	0.55285 (15)	0.81503 (14)	0.0260 (3)	
H24A	0.9624	0.5680	0.9024	0.031*	
H24B	0.8381	0.4734	0.7814	0.031*	
C25	1.02476 (16)	0.51780 (16)	0.73060 (14)	0.0311 (4)	
H25A	0.9762	0.4906	0.6411	0.037*	
H25B	1.0966	0.6017	0.7571	0.037*	
C26	1.09912 (18)	0.40151 (16)	0.73654 (16)	0.0359 (4)	
H26A	1.1529	0.4301	0.8237	0.054*	
H26B	1.1637	0.3809	0.6775	0.054*	
H26C	1.0286	0.3185	0.7122	0.054*	

### Atomic displacement parameters (Å^2^)

**Table:**

	*U*^11^	*U*^22^	*U*^33^	*U*^12^	*U*^13^	*U*^23^
C1	0.0207 (7)	0.0229 (7)	0.0170 (7)	0.0013 (6)	0.0033 (6)	0.0072 (6)
C2	0.0205 (7)	0.0251 (7)	0.0170 (7)	0.0031 (6)	0.0037 (5)	0.0097 (6)
N2	0.0241 (6)	0.0216 (6)	0.0168 (6)	0.0025 (5)	0.0030 (5)	0.0075 (5)
C3	0.0221 (7)	0.0216 (7)	0.0172 (7)	0.0025 (6)	0.0059 (6)	0.0071 (6)
N1	0.0229 (6)	0.0238 (6)	0.0180 (6)	0.0035 (5)	0.0047 (5)	0.0081 (5)
C4	0.0243 (7)	0.0244 (7)	0.0154 (7)	0.0067 (6)	0.0074 (6)	0.0070 (6)
C5	0.0268 (8)	0.0255 (8)	0.0196 (7)	0.0052 (6)	0.0032 (6)	0.0066 (6)
C6	0.0352 (9)	0.0212 (7)	0.0255 (8)	0.0044 (6)	0.0090 (7)	0.0083 (6)
C7	0.0373 (9)	0.0294 (8)	0.0235 (8)	0.0136 (7)	0.0083 (7)	0.0124 (7)
C8	0.0292 (8)	0.0331 (8)	0.0209 (7)	0.0088 (7)	0.0034 (6)	0.0098 (7)
C9	0.0259 (8)	0.0235 (8)	0.0211 (7)	0.0045 (6)	0.0054 (6)	0.0067 (6)
C10	0.0229 (7)	0.0201 (7)	0.0182 (7)	0.0053 (6)	0.0031 (6)	0.0067 (6)
C11	0.0296 (8)	0.0271 (8)	0.0222 (7)	0.0021 (6)	0.0084 (6)	0.0056 (6)
C12	0.0265 (8)	0.0299 (8)	0.0327 (9)	−0.0018 (7)	0.0048 (7)	0.0047 (7)
C13	0.0321 (9)	0.0293 (8)	0.0208 (7)	0.0055 (7)	−0.0042 (6)	0.0038 (6)
C14	0.0405 (9)	0.0363 (9)	0.0184 (7)	0.0037 (7)	0.0042 (7)	0.0096 (7)
C15	0.0292 (8)	0.0305 (8)	0.0223 (7)	−0.0008 (6)	0.0031 (6)	0.0116 (7)
C16	0.0223 (7)	0.0227 (7)	0.0187 (7)	0.0009 (6)	0.0033 (6)	0.0089 (6)
C17	0.0254 (8)	0.0257 (8)	0.0177 (7)	0.0023 (6)	0.0049 (6)	0.0065 (6)
C18	0.0225 (8)	0.0286 (8)	0.0238 (7)	0.0046 (6)	0.0053 (6)	0.0114 (6)
C19	0.0217 (7)	0.0316 (8)	0.0189 (7)	−0.0009 (6)	0.0008 (6)	0.0128 (6)
O119	0.0271 (6)	0.0383 (6)	0.0204 (5)	0.0066 (5)	0.0007 (4)	0.0135 (5)
C119	0.0310 (9)	0.0456 (10)	0.0290 (8)	0.0105 (8)	−0.0007 (7)	0.0180 (8)
C20	0.0277 (8)	0.0267 (8)	0.0185 (7)	0.0024 (6)	0.0055 (6)	0.0064 (6)
C21	0.0243 (8)	0.0255 (7)	0.0210 (7)	0.0040 (6)	0.0048 (6)	0.0084 (6)
C22	0.0249 (7)	0.0206 (7)	0.0214 (7)	0.0022 (6)	0.0013 (6)	0.0092 (6)
C23	0.0268 (8)	0.0234 (7)	0.0188 (7)	0.0033 (6)	0.0026 (6)	0.0068 (6)
C24	0.0276 (8)	0.0263 (8)	0.0227 (7)	0.0054 (6)	0.0037 (6)	0.0095 (6)
C25	0.0320 (9)	0.0300 (8)	0.0292 (8)	0.0073 (7)	0.0080 (7)	0.0097 (7)
C26	0.0339 (9)	0.0317 (9)	0.0369 (9)	0.0088 (7)	0.0076 (7)	0.0091 (7)

### Geometric parameters (Å, º)

**Table:**

C1—C2	1.3721 (19)	C16—C17	1.390 (2)
C1—N1	1.3811 (17)	C16—C21	1.4045 (19)
C1—C4	1.4716 (19)	C17—C18	1.3864 (19)
C2—N2	1.3833 (17)	C17—H17	0.9500
C2—C10	1.4848 (18)	C18—C19	1.386 (2)
N2—C3	1.3742 (17)	C18—H18	0.9500
N2—C22	1.4622 (17)	C19—O119	1.3724 (16)
C3—N1	1.3188 (17)	C19—C20	1.393 (2)
C3—C16	1.4730 (19)	O119—C119	1.4272 (18)
C4—C9	1.3950 (19)	C119—H11A	0.9800
C4—C5	1.396 (2)	C119—H11B	0.9800
C5—C6	1.386 (2)	C119—H11C	0.9800
C5—H5	0.9500	C20—C21	1.3772 (19)
C6—C7	1.389 (2)	C20—H20	0.9500
C6—H6	0.9500	C21—H21	0.9500
C7—C8	1.381 (2)	C22—C23	1.529 (2)
C7—H7	0.9500	C22—H22A	0.9900
C8—C9	1.384 (2)	C22—H22B	0.9900
C8—H8	0.9500	C23—C24	1.5225 (19)
C9—H9	0.9500	C23—H23A	0.9900
C10—C15	1.389 (2)	C23—H23B	0.9900
C10—C11	1.389 (2)	C24—C25	1.521 (2)
C11—C12	1.392 (2)	C24—H24A	0.9900
C11—H11	0.9500	C24—H24B	0.9900
C12—C13	1.378 (2)	C25—C26	1.521 (2)
C12—H12	0.9500	C25—H25A	0.9900
C13—C14	1.375 (2)	C25—H25B	0.9900
C13—H13	0.9500	C26—H26A	0.9800
C14—C15	1.384 (2)	C26—H26B	0.9800
C14—H14	0.9500	C26—H26C	0.9800
C15—H15	0.9500		
			
C2—C1—N1	110.31 (12)	C18—C17—C16	121.84 (13)
C2—C1—C4	129.52 (12)	C18—C17—H17	119.1
N1—C1—C4	120.10 (12)	C16—C17—H17	119.1
C1—C2—N2	105.42 (11)	C19—C18—C17	119.52 (14)
C1—C2—C10	132.42 (13)	C19—C18—H18	120.2
N2—C2—C10	121.98 (12)	C17—C18—H18	120.2
C3—N2—C2	107.27 (11)	O119—C19—C18	124.04 (13)
C3—N2—C22	127.52 (12)	O119—C19—C20	116.28 (12)
C2—N2—C22	125.14 (11)	C18—C19—C20	119.63 (13)
N1—C3—N2	111.01 (12)	C19—O119—C119	117.07 (11)
N1—C3—C16	123.28 (12)	O119—C119—H11A	109.5
N2—C3—C16	125.62 (12)	O119—C119—H11B	109.5
C3—N1—C1	105.98 (11)	H11A—C119—H11B	109.5
C9—C4—C5	118.02 (13)	O119—C119—H11C	109.5
C9—C4—C1	122.72 (13)	H11A—C119—H11C	109.5
C5—C4—C1	119.25 (13)	H11B—C119—H11C	109.5
C6—C5—C4	121.02 (13)	C21—C20—C19	120.41 (13)
C6—C5—H5	119.5	C21—C20—H20	119.8
C4—C5—H5	119.5	C19—C20—H20	119.8
C5—C6—C7	119.98 (14)	C20—C21—C16	120.87 (14)
C5—C6—H6	120.0	C20—C21—H21	119.6
C7—C6—H6	120.0	C16—C21—H21	119.6
C8—C7—C6	119.63 (14)	N2—C22—C23	112.03 (12)
C8—C7—H7	120.2	N2—C22—H22A	109.2
C6—C7—H7	120.2	C23—C22—H22A	109.2
C7—C8—C9	120.34 (14)	N2—C22—H22B	109.2
C7—C8—H8	119.8	C23—C22—H22B	109.2
C9—C8—H8	119.8	H22A—C22—H22B	107.9
C8—C9—C4	121.00 (14)	C24—C23—C22	113.10 (12)
C8—C9—H9	119.5	C24—C23—H23A	109.0
C4—C9—H9	119.5	C22—C23—H23A	109.0
C15—C10—C11	118.59 (13)	C24—C23—H23B	109.0
C15—C10—C2	119.43 (13)	C22—C23—H23B	109.0
C11—C10—C2	121.97 (13)	H23A—C23—H23B	107.8
C10—C11—C12	120.06 (14)	C25—C24—C23	112.34 (12)
C10—C11—H11	120.0	C25—C24—H24A	109.1
C12—C11—H11	120.0	C23—C24—H24A	109.1
C13—C12—C11	120.69 (15)	C25—C24—H24B	109.1
C13—C12—H12	119.7	C23—C24—H24B	109.1
C11—C12—H12	119.7	H24A—C24—H24B	107.9
C14—C13—C12	119.50 (14)	C26—C25—C24	113.26 (14)
C14—C13—H13	120.2	C26—C25—H25A	108.9
C12—C13—H13	120.2	C24—C25—H25A	108.9
C13—C14—C15	120.24 (15)	C26—C25—H25B	108.9
C13—C14—H14	119.9	C24—C25—H25B	108.9
C15—C14—H14	119.9	H25A—C25—H25B	107.7
C14—C15—C10	120.91 (15)	C25—C26—H26A	109.5
C14—C15—H15	119.5	C25—C26—H26B	109.5
C10—C15—H15	119.5	H26A—C26—H26B	109.5
C17—C16—C21	117.68 (13)	C25—C26—H26C	109.5
C17—C16—C3	124.27 (12)	H26A—C26—H26C	109.5
C21—C16—C3	118.03 (13)	H26B—C26—H26C	109.5

### Hydrogen-bond geometry (Å, º)

**Table:**

*D*—H···*A*	*D*—H	H···*A*	*D*···*A*	*D*—H···*A*
C15—H15···O119^i^	0.95	2.61	3.4393 (19)	146

Symmetry code: (i) −*x*+2, −*y*+2, −*z*+2.
